# Cost-Effectiveness of Gliclazide-Based Intensive Glucose Control vs. Standard Glucose Control in Type 2 Diabetes Mellitus. An Economic Analysis of the ADVANCE Trial in Vietnam

**DOI:** 10.3389/fpubh.2020.562023

**Published:** 2020-10-30

**Authors:** Hai-Yen Nguyen-Thi, Nga TQ. Nguyen, Nguyen Dang Tu Le, Maud Beillat, Olivier Ethgen

**Affiliations:** ^1^Department of Pharmaceutical Administration, Faculty of Pharmacy, University of Medicine and Pharmacy at Ho Chi Minh City, Ho Chi Minh City, Vietnam; ^2^Centre for Public Health, School of Medicine, Dentistry and Biomedical Sciences, Queen's University Belfast, Belfast, United Kingdom; ^3^Servier Global Market Access & Health Economics and Outcomes Research, Suresnes, France; ^4^SERFAN Innovation, Namur, Belgium; ^5^Department of Public Health, Epidemiology and Health Economics, University of Liège, Liège, Belgium

**Keywords:** gliclazide, intensive glucose control, type 2 diabetes mellitus, hyperglycemia, end-stage renal disease, cost-effectiveness, Vietnam

## Abstract

**Introduction:** ADVANCE was a large, multinational clinical study conducted over 5 years in type 2 diabetes mellitus (T2DM). In all, 11,140 patients were randomly assigned to receive gliclazide-based intensive glucose control (IGC) or standard glucose control (SGC). IGC was shown to significantly reduce the incidence of major macrovascular and microvascular events (composite endpoint) or major microvascular events compared with SGC, primarily by enhancing renal protection. We assessed the cost-effectiveness of IGC vs. SGC, based on the ADVANCE results, from a Vietnamese healthcare payer perspective.

**Materials and Methods:** A partitioned survival times model across five health states (no complications, myocardial infarction, stroke, end-stage renal disease [ESRD], and diabetes-related eye-disease) was designed. Time-to-event curves were informed by the cumulative incidence of events and corresponding hazard ratios from the ADVANCE study. Health outcomes were expressed in terms of ESRD avoided and quality-adjusted life years (QALYs). Costs (in US $) comprised treatment costs and health state costs. Utility weights and costs were documented from literature reporting Vietnamese estimates. For sensitivity analyses, all parameters were individually varied within their 95% confidence interval bounds (when available) or within a ±30% range.

**Results:** Over a 5-year horizon, IGC avoided 6.5 additional ESRD events per 1,000 patients treated compared with SGC (IGC, 3.5 events vs. SGC, 10.0 events) and provided 0.016 additional QALYs (IGC, 3.570 QALYs vs. SGC, 3.555 QALYs). Total costs were similar for the two strategies (IGC, $3,786 vs. SGC, $3,757). Although the total drug costs were markedly higher for IGC compared with SGC ($1,703 vs. $873), this was largely offset by the savings from better renal protection with IGC (IGC, $577 vs. SGC, $1,508). The incremental cost-effectiveness ratio (ICER) of IGC vs. SGC was $1,878/QALY gained, far below the threshold recommended by the World Health Organization (i.e., 1–3 × gross domestic product per inhabitant ≈$7,500 in Vietnam). The ICER of IGC vs. SGC per ESRD event avoided was $4,559/event. The findings were robust to sensitivity analysis.

**Conclusion:** In Vietnam, gliclazide-based IGC was shown to be cost-effective compared with SGC from a healthcare payer perspective, as defined in the ADVANCE study.

## Introduction

Worldwide, over 400 million individuals have diabetes; 90% of these have type 2 diabetes mellitus (T2DM) (1). In particular, increased consumption of unhealthy diets high in red or processed meat, refined grains, and sugar-sweetened beverages have been key to the increasing prevalence of T2DM, and the recent rapid transition to such diets has been linked to the increasing prevalence of T2DM in Asia (1). Asian populations appear to be at higher risk of developing T2DM than other ethnic groups. For example, data from the USA have shown that people of Asian descent are 30–35% more likely to develop T2DM than non-Hispanic whites, despite having lower body mass index (2). The prevalence of T2DM in Vietnam is increasing rapidly, as evidenced by an estimated doubling in national prevalence within 10 years (from 2.7% in 2002 to 5.4% in 2012) (3). This increase has consequently created a considerable and growing economic burden in Vietnam (4). For instance, the estimated annual cost per patient with T2DM was 246.10 US dollars, which equates to around 12% of gross domestic product per capita in 2017 (5). Therefore, a clear need exists to reduce the economic impact of the disease.

Treatment of T2DM focuses on the attainment of good glycemic control. Metformin is the first-line medication, combined with lifestyle changes covering diet and exercise (6, 7). Second-line medications include sulphonylureas, a class of drugs that induce glucose-independent insulin secretion (8). Sulphonylureas have been in use for T2DM for decades and their efficacy is well-established (9). They also remain a lower cost option than newer second-line non-insulin agents (6, 7), and real-world data indicate that they are used in a large number of T2DM patients (10). Consequently, sulphonylureas are still the main second-line treatment globally, despite the emergence of newer classes of drugs for glycemic control (11–13). Gliclazide is a sulphonylurea that has been shown to have a better safety profile than other drugs in its class (9). As such, gliclazide remains an important component of the T2DM treatment pathway (8).

In diabetes, hyperglycemia is strongly linked with micro- and macrovascular complications. Microvascular complications include conditions such as retinopathy, neuropathy, and diabetic nephropathy, while macrovascular complications arise from the formation of atherosclerotic plaques in major blood vessels, leading to outcomes such as myocardial infarction and stroke (14). Current diabetes guidelines recommend a target glycated hemoglobin level ≤ 7.0% (7); however, there is evidence that intensive glucose control (IGC) regimes may offer benefits to patients, particularly with regard to reducing the risk of microvascular complications (15). The Action in Diabetes and Vascular Disease: Preterax and Diamicron Modified Release Controlled Evaluation (ADVANCE) trial was a global, randomized, controlled trial designed to assess the effects of IGC on major vascular outcomes in a broad cross-section of patients with T2DM. Patients were randomized to standard glucose control (SGC, *n* = 5,569) or IGC (*n* = 5,571), defined as the use of gliclazide modified release plus other drugs as required to achieve a glycated hemoglobin level ≤ 6.5%, and were followed-up for a median duration of 5.0 years (16). IGC significantly reduced the risk of combined major macro- and microvascular events (composite endpoint) compared with SGC, largely driven by a 21% relative reduction in the incidence of nephropathy (16, 17). A significant reduction in the risk of end-stage renal disease (ESRD) was also observed with IGC compared with SGC during the in-trial period, which persisted during long-term (total 9.9 years) follow-up (18).

With the rapidly increasing prevalence of T2DM in Asia (and notably Vietnam), and the associated economic burden, it is essential that the most cost-effective interventions are identified. The objective of this analysis is to assess the cost-effectiveness of IGC vs. SGC from a Vietnamese healthcare payer perspective, using clinical outcomes identified from the ADVANCE trial.

## Methods

### Model Structure

**Figure 1:**
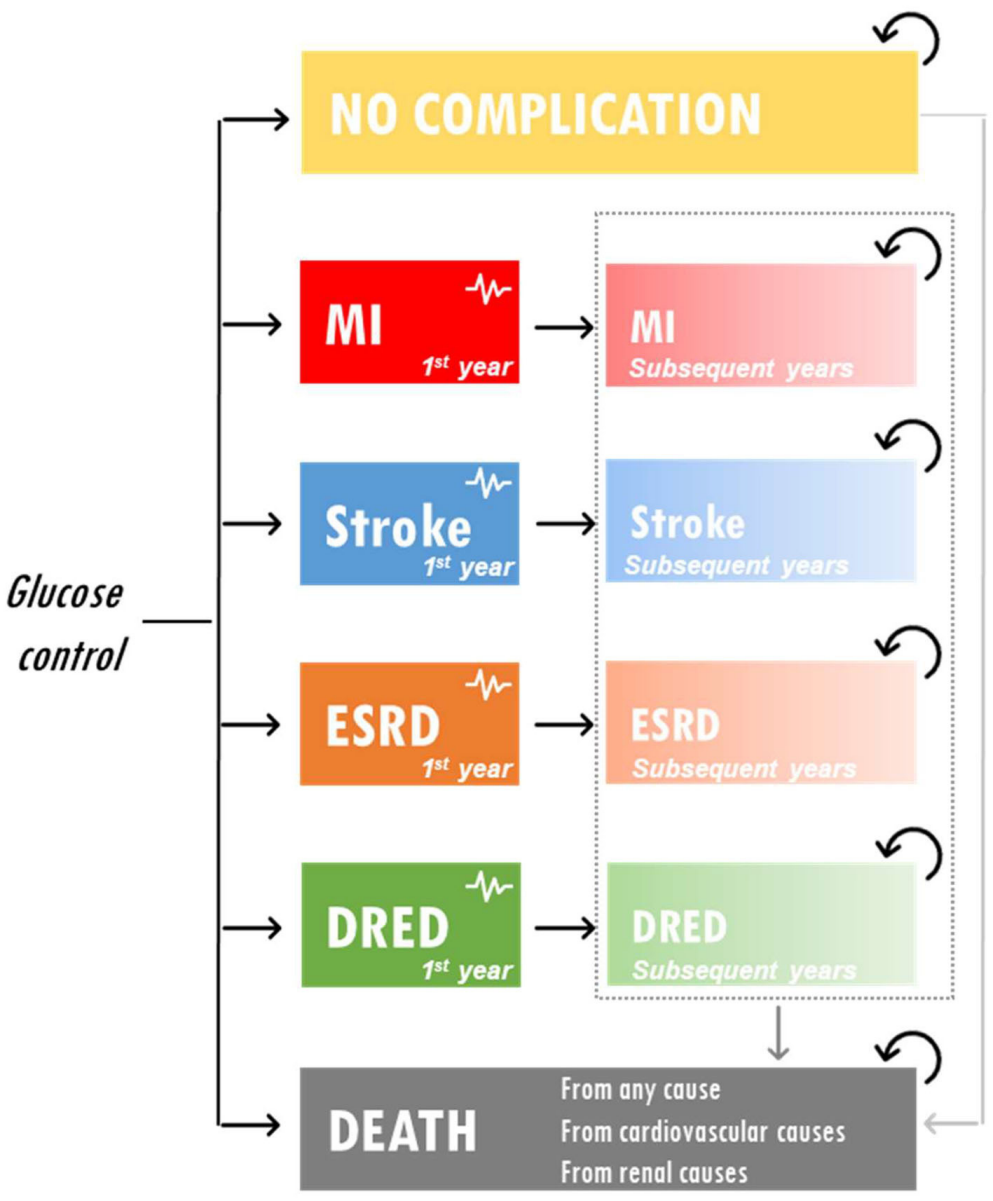
Model schematic. DRED, diabetes-related eye disease; ESRD, end-stage renal disease; MI, myocardial infarction.

A partitioned survival model was developed to assess the cost-effectiveness of IGC vs. SGC in Vietnam. The model included five health states representative of T2DM complications and reflecting the key end points included in the ADVANCE trial: no complications, myocardial infarction, stroke, ESRD, and diabetes-related eye disease. The model structure is presented in Figure 1. Costs were reported in US dollars and health outcomes were expressed in terms of life years (LYs), quality-adjusted life years (QALYs), and ESRD events avoided. The model had a time horizon of 5 years to be aligned with ADVANCE. A discount rate of 3% was applied to both costs and outcomes.

### Efficacy and Safety Outcomes

Efficacy and safety outcomes for the IGC and SGC treatment approaches were derived from the ADVANCE study (16). For each complication, time-to-event curves were informed by the cumulative incidence of events and corresponding hazard ratios. Baseline patient characteristics and clinical inputs included in the model are presented in Tables 1, 2.

**Table 1:** Patient characteristics at baseline in the ADVANCE trial.

	**IGC**	**SGC**
	**(*N* = 5,571)**	**(*N* = 5,569)**
Mean (±SD) age, years	66 ± 6	66 ± 6
Female sex, *n* (%)	2,376 (42.6)	2,357 (42.3)
Mean (±SD) age when diabetes first diagnosed, year	58 ± 9	58 ± 9
Mean (±SD) duration of diabetes, years	7.9 ± 6.3	8.0 ± 6.4
Mean (±SD) standardized glycated hemoglobin^*^, %	7.48 ± 1.65	7.48 ± 1.63

* *Laboratories participating in ADVANCE underwent a standardization process using the Wales External Quality Assurance Scheme. Source (16)*.

**Table 2:** Clinical inputs included in the model.

	**Base case**	DSA lower value^*^	DSA upper value^*^	**PSA distribution**	**Source**
**Deaths from any cause**					
Standard	9.6%	6.7%	12.5%	Beta	Advance Collaborative Group NEJM 2008 (16)
HR_Intensivevs.standard^**^_	0.93	0.83	1.06	Log-normal	Advance-ON Collaborative Group NEJM 2014 (19)
**Major macrovascular events**					
Standard	10.6%	7.4%	13.8%	Beta	Advance Collaborative Group NEJM 2008 (16)
HR_Intensive vs. standard_	0.94	0.84	1.06	Log-normal	Advance-ON Collaborative Group NEJM 2014 (19)
**Death from CV causes**					
Standard	5.2%	3.6%	6.8%	Beta	Advance Collaborative Group NEJM 2008 (16)
HR_Intensive vs. standard_	0.88	0.74	1.04	Log-normal	Advance-ON Collaborative Group NEJM 2014 (19)
**MI**					
Standard	3.4%	2.4%	4.4%	Beta	Advance Collaborative Group NEJM 2008 (16)
HR_Intensive vs. standard_	1.01	0.83	1.24	Log-normal	Advance-ON Collaborative Group NEJM 2014 (19)
**Stroke**					
Standard	4.4%	3.1%	5.7%	Beta	Advance Collaborative Group NEJM 2008 (16)
HR_Intensive vs. standard_	0.96	0.81	1.15	Log-normal	Advance-ON Collaborative Group NEJM 2014 (19)
**Major microvascular events**					
Standard	4.4%	3.1%	5.7%	Beta	Advance Collaborative Group NEJM 2008 (16)
HR_Intensive vs. standard_	0.86	0.72	1.03	Log-normal	Advance-ON Collaborative Group NEJM 2014 (19)
**ESRD**					
Standard	1.0%	0.7%	1.3%	Beta	Advance Collaborative Group NEJM 2008 (16)
HR_Intensive vs. standard_	0.35	0.15	0.83	Log-normal	Advance-ON Collaborative Group NEJM 2014 (19)
**Death from renal causes**					
Standard	0.4%	0.3%	0.5%	Beta	Advance Collaborative Group NEJM 2008 (16)
HR_Intensive vs. standard_	0.85	0.45	1.62	Log-normal	Advance-ON Collaborative Group NEJM 2014 (19)
**DRED**					
Standard	3.9%	2.7%	5.1%	Beta	Advance Collaborative Group NEJM 2008 (16)
HR_Intensive vs. standard_	0.90	0.74	1.09	Log-normal	Advance-ON Collaborative Group NEJM 2014 (19)
**Major hypoglycemia**					
Standard	1.5%	1.1%	2.0%	Beta	Advance Collaborative Group NEJM 2008 (16)
HR_Intensive vs. standard_	1.85	1.42	2.42	Log-normal	Advance-ON Collaborative Group NEJM 2014 (19)

* ±30% margins.

** 95% confidence interval as reported in the ADVANCE study.

*CV, cardiovascular; DRED, diabetes-related eye disease; DSA, deterministic sensitivity analysis; ESRD, end-stage renal disease; HR, hazard ratio; MI, myocardial infarction; PSA, probabilistic sensitivity analysis*.

### Costs and Health Utilities

Costs were estimated from a national healthcare payer perspective and included treatment costs and health state costs. In the absence of reliable Vietnamese estimates, health state costs were derived from published data in Thailand (20) using purchasing power parity exchange rates (21). Cost of death was assumed to be zero (regardless of cause), and drug costs were taken from Vietnamese sources. The Vietnamese national insurance payer reimburses medication costs on a case-by-case basis (22), and drug pricing in each healthcare unit is based on the procurement price published by the Ministry of Health (Drug Administration of Vietnam) or Social Insurance Office in the last 12 months (23). Therefore, drug costs were retrieved from the procurement price list published by the Drug Administration of Vietnam (latest version published September 2018).

In order to adjust LYs gained to produce the outcome of QALYs in the model, health-related quality of life data were required to determine the impact of different health outcomes on patient utility. Utility values are measured on an interval scale with 0 reflecting death and 1 reflecting perfect health. As the ADVANCE study did not evaluate health-related quality of life, utility weights were derived from published health-related quality of life estimates using the EQ-5D-5L instrument in Vietnam (24). Disutility weights were obtained from previous cost-effectiveness studies, notably the Core Diabetes Model (25). All costs and health state utilities included in the model are presented in Table 3. For costs and utilities, a distinction was made between the year of occurrence and subsequent year for each health state.

**Table 3:** Summary of costs and utilities included in the model.

	**Base case**	DSA lower value^*^	DSA upper value^*^	**PSA distribution**	**Source**
**Annual costs of drug regimen (USD)**					
*Standard glucose control*	164	115	319	Gamma	Procurement price list, Drug Administration of Vietnam
*Intensive glucose control*	319	223	415	Gamma	Procurement price list, Drug Administration of Vietnam
**Health state costs (USD 2014)**					
*Myocardial infarction* 1st year Subsequent years	14,975 3,751	10,483 2,626	19,468 4,876	Gamma Gamma	Permsuwan et al. (20)
*Stroke* 1st year Subsequent years	10,051 3,364	7,036 2,355	13,066 4,373	Gamma Gamma	Permsuwan et al. (20)
*ESRD* 1st year Subsequent years	86,397 59,411	60,478 41,588	112,316 77,234	Gamma Gamma	Permsuwan et al. (20)
*DRED* 1st year Subsequent years	4,352 2,643	3,046 1,850	5,658 3,436	Gamma Gamma	Permsuwan et al. (20)
*Major hypoglycemia* Per event	3,885	2,720	5,051	Gamma	Permsuwan et al. (20)
**Health state utilities**					
Population norms Baseline age (i.e., 66 years) 5 years after (i.e., 71 years)	0.810 0.808	0.567 0.566	1.000 0.810	Beta Beta	Nguyen et al. (24)
**Events disutilities (%)**					
*Myocardial infarction* 1st year Subsequent years	−15.9 −9.6	−11.1 −6.7	−20.6 −12.5	Beta Beta	Palmer et al. (25)
*Stroke* 1st year Subsequent years	−22.2 −33.1	−15.6 −23.1	−28.9 −43.0	Beta Beta	Palmer et al. (25)
*ESRD* 1st year Subsequent years	−35.5 −35.5	−24.9 −24.9	−46.2 −46.2	Beta Beta	Palmer et al. (25)
*DRED* 1st year Subsequent years	−9.8 −9.8	−6.9 −6.9	−12.8 −12.8	Beta Beta	Palmer et al. (25)
*Major hypoglycemia* Per event	−0.6	−0.4	−0.8	Beta	Palmer et al. (25)

* ±30% margins.

*CV, cardiovascular; DRED, diabetes-related eye disease; DSA, deterministic sensitivity analysis; ESRD, end-stage renal disease; PSA, probabilistic sensitivity analysis; USD, United States Dollars*.

### Sensitivity Analyses

Uncertainty surrounding input parameter values was addressed by conducting deterministic sensitivity analyses in which one input parameter value was varied at a time. Base-case values were varied within their 95% confidence interval bounds (when available) or within a ±30% range. Probabilistic sensitivity analyses were also performed in order to determine the impact of parameter uncertainty on the outcomes of the model. In this analysis, multiple parameter values were varied simultaneously and a Monte Carlo simulation was run (1,000 iterations). For utility and proportions, a Beta distribution was assumed, whereas hazard ratios were assumed to follow a Log-Normal distribution and costs a Gamma distribution (26).

## Results

### Base-Case Analysis

The results of the base-case analysis are presented in Table 4. Over 5 years, IGC resulted in the avoidance of 6.5 additional ESRD events per 1,000 patients treated compared with SGC (3.5 events vs. 10 events, respectively). The incremental LYs and QALYs were 0.017 and 0.016, respectively, for IGC compared with SGC (LYs; IGC, 4.777 vs. SGC, 4.760: QALYs; IGC, 3.570 vs. SGC, 3.555). Although the treatment costs were higher for IGC ($1,703) than SGC ($873), this was largely offset by the savings from the reduced number of ESRD events with IGC ($577) vs. SGC ($1,508). This resulted in very similar total costs between the two strategies (IGC, $3,786 vs. SGC, $3,757). The resulting incremental cost-effectiveness ratios (ICERs) were $4,559, $1,764, and $1,878 per ESRD event avoided, LY gained, and QALY gained, respectively.

**Table 4:** Base-case model results.

	**Absolute**		**Incremental**	
	**Standard**	**Intensive**		**% change**
**ESRD event (per 1,000 patients)**	10.0	3.5	−6.5	−65.0%
**LYs**	4.760	4.777	0.017	+0.4%
**QALYs**	3.555	3.570	0.016	+0.4%
**Costs (USD)** Treatment MI Stroke ESRD DRED Hypoglycemic event Death	3,757 873 480 463 1,508 276 157 0	3,786 1,703 493 451 577 272 290 0	30 831 13 −11 −931 −5 133 0	+0.8% +95.2% +2.6% −2.4% −61.7% −1.6% +85.0% NA
**ICER ESRD avoided**			4,559	
**ICER LY**			1,764	
**ICER QALY**			1,878	

*DRED, diabetes-related eye disease; ESRD, end-stage renal disease; ICER, incremental cost-effectiveness ratio; LY, life year; MI, myocardial infarction; NA, not applicable; QALY, quality-adjusted life year; USD, United States Dollars*.

### Deterministic Sensitivity Analyses

**Figure 2:**
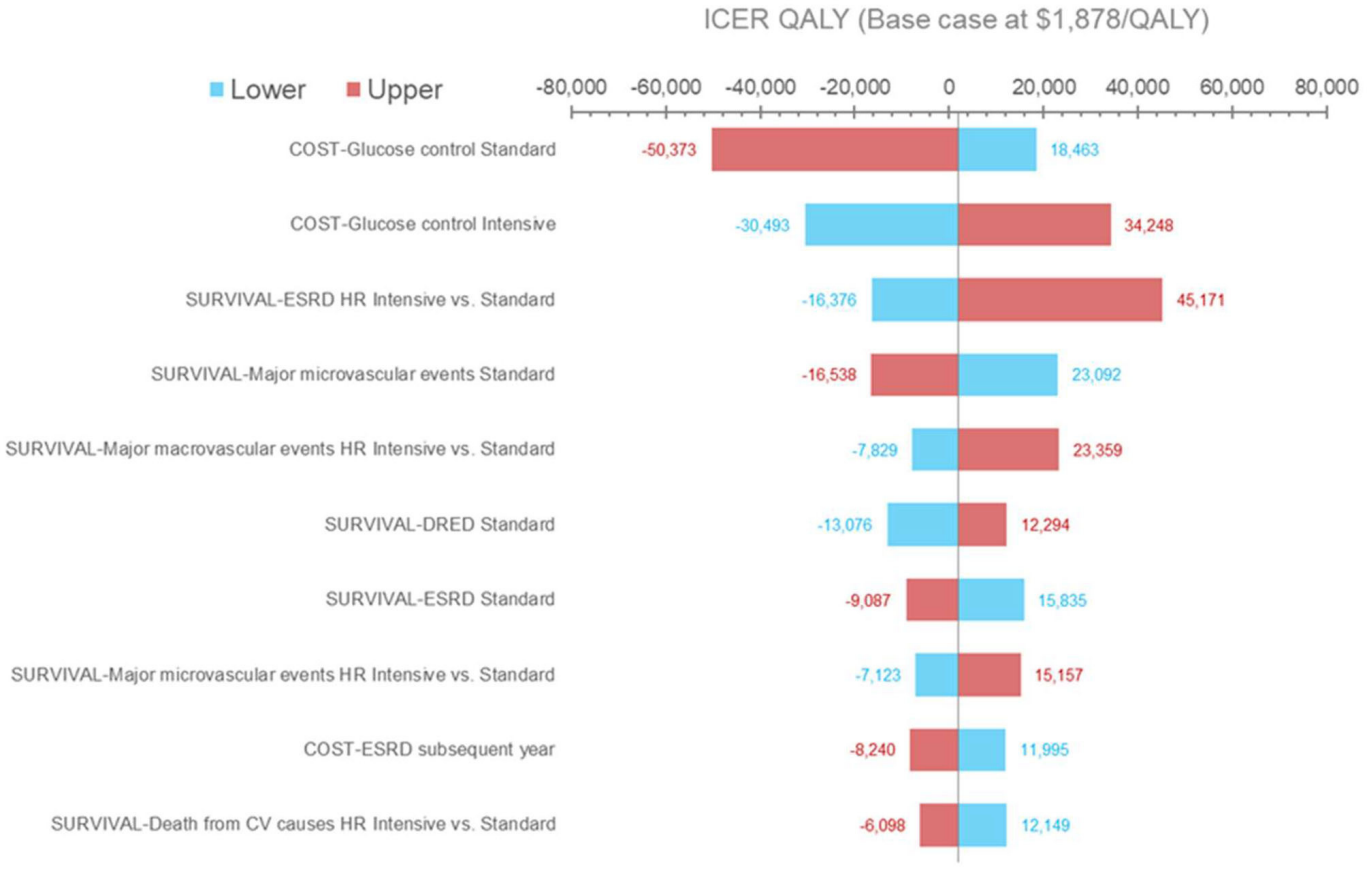
Deterministic sensitivity analysis results (QALYs). Results of the one-way sensitivity analysis in which several model input parameters were varied to determine their effect on output. Blue bars and red bars represent the lower and upper bound of each parameter varied, respectively. The horizontal axis represents the incremental cost-effectiveness ratio value per QALY gained. The vertical center line represents the base case. CV, cardiovascular; DRED, diabetes-related eye disease; ESRD, end-stage renal disease; HR, hazard ratio; ICER, incremental cost-effectiveness ratio; QALY, quality-adjusted life year.

Results from the deterministic sensitivity analysis are presented in Figure 2. The ICER was most sensitive to variations in treatment costs and clinical outcomes, most notably with variations in the risk of experiencing ESRD events. Using the lower range of treatment costs for SGC or the upper range of treatment costs for IGC increased the ICER per QALY gained to $18,463 and $34,248, respectively. Applying the upper range of the treatment costs for SGC or the lower range of the treatment costs for IGC would lead to IGC becoming the dominant strategy. Using the lower range of the hazard ratio (IGC vs. SGC) for ERSD events (i.e., assuming that IGC is more effective than in the base-case analysis) would result in IGC becoming dominant, whereas using the higher range of the hazard ratio (i.e., assuming that IGC is less effective than the base-case analysis) increased the ICER per QALY gained to $45,171. The ICER did not change substantially across the other parameters tested (data not shown), including health state utilities, discounting rates, and most health state costs (with the exception of costs associated with ESRD events and major hypoglycemic events).

### Probabilistic Sensitivity Analyses

**Figure 3:**
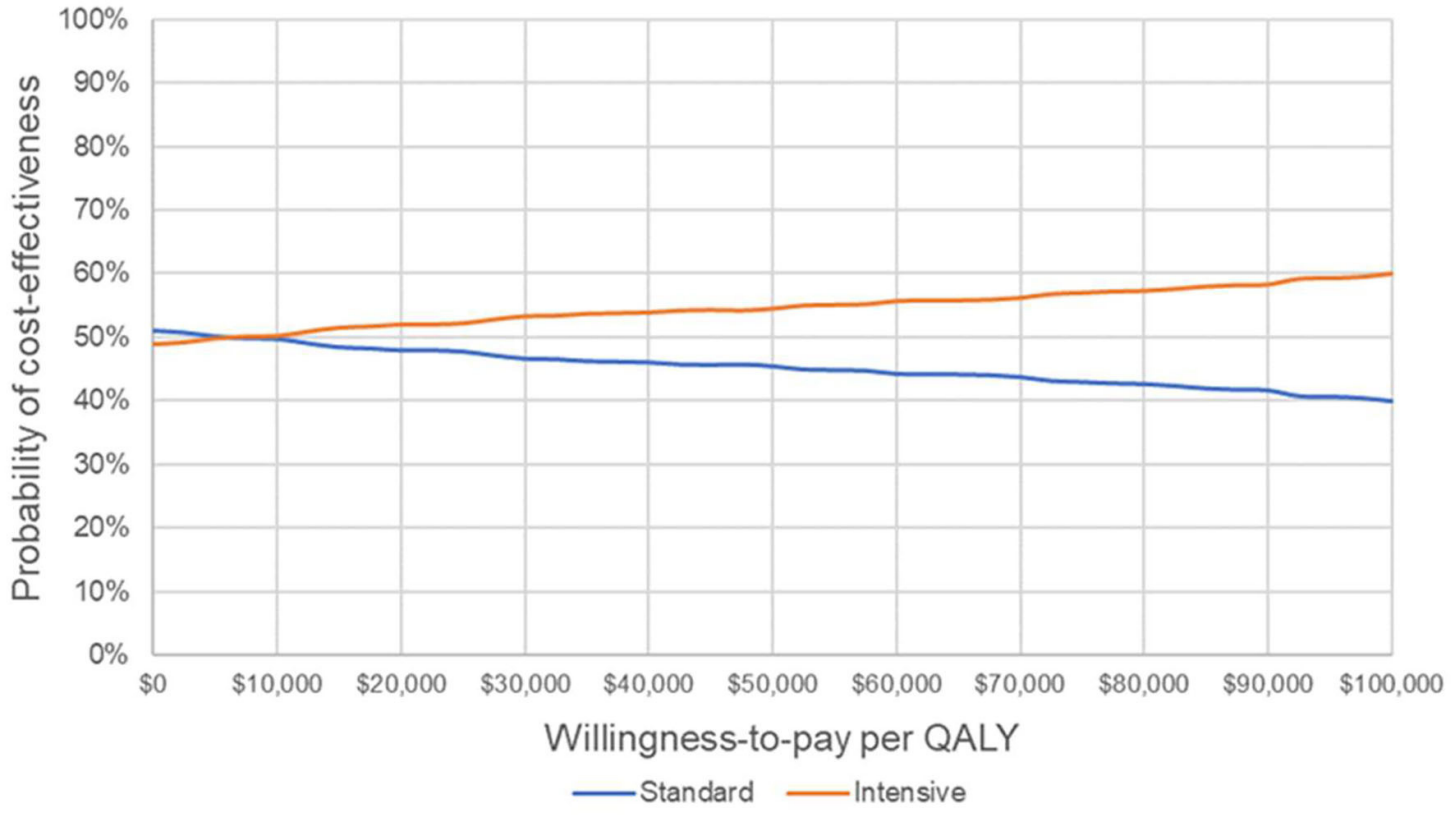
Cost-effectiveness acceptability curve. Probability that each strategy is cost-effective at varying willingness-to-pay thresholds. QALY, quality-adjusted life year.

The results of the probabilistic sensitivity analysis are shown in Figure 3. The cost-effectiveness acceptability curve suggests that IGC becomes the strategy with the highest probability of cost-effectiveness from a willingness-to-pay threshold of ~$7,000 per QALY.

## Discussion

The results of this cost-effectiveness analysis indicate that IGC is cost-effective in Vietnam compared with SGC. The ICERs/QALY gained for the base-case analysis compare favorably with the World Health Organization cost-effectiveness threshold for intervention, which at 1–3 times gross domestic product per capita for intervention (27) is ~$7,500 in Vietnam [2018 value; (28)]. As a result of the increasing prevalence of T2DM in Vietnam (3), and the considerable and rising economic burden it creates (4), more widespread adoption of effective and cost-effective interventions could have a substantial public health impact. However, knowledge and awareness of T2DM among the general population of Vietnam and similar countries is low (29) and, compliance with treatment and awareness of the importance of diabetes control requires considerable improvement (30). Therefore, disease awareness campaigns and education programs are required alongside effective pharmacotherapies to maximize the potential public health benefit.

There is increasing demand for more efficient allocation of scarce healthcare resources. This demand is particularly great in highly prevalent chronic diseases such as T2DM, due to their potential impact on patient quality of life and healthcare expenditure. Vietnam is in the early stages of adopting health technology assessments to guide decisions on allocation of healthcare resources (31, 32). Therefore, analyses such as the current one will be an important component of health technology assessments needed to support policymakers with decision-making.

The IMS CORE Diabetes Model (CDM) is a widely published and validated simulation model applied to type 1 and T2DM (33). The CDM is used to estimate long-term health and economic outcomes for populations, accounting for detailed past history, disease management and physiological parameters. We therefore developed a partitioned survival model as a more appropriate way to conduct a trial-based cost-effectiveness analysis. The economic analysis presented here has a number of strengths, but also some limitations. Strengths include the fact that it utilizes clinical data from a large, randomized, controlled trial conducted across many countries (with a wide variety of health systems and incomes). In addition, scenario analyses showed that the model used is highly sensitive to variations in treatment costs and clinical outcomes, demonstrating that the model has high internal validity. Furthermore, the adaptation process was thorough and followed published methodological recommendations (34). A potential limitation of the current analysis is that the comparisons and model were based on 5-year data (the duration of the ADVANCE study). No extrapolation beyond 5 years was included, due to the high level of uncertainty involved (for example, with respect to treatments received beyond the in-trial 5-year follow-up, and also an absence of data on adherence/persistence in Vietnam). Furthermore, less than half of patients in ADVANCE were from the Asian region (and none of the included patients were from Vietnam), and adaption to the Vietnamese population required use of estimates based on published literature. Nevertheless, sensitivity analyses support the utility of the model used. The comparison used in the model also omitted newer classes of drugs available for the management of T2DM (including sodium-glucose co-transporter-2 inhibitors, dipeptidyl peptidase-4 inhibitors, and glucagon-like peptide 1 receptor agonists), which have been shown to provide benefits (35). However, use of these newer drugs may be limited by affordability and accessibility (7). In addition, data from a multi-center study in Vietnam indicate that sulphonylureas are much more widely used for glycemic control (55% of patients) than dipeptidyl peptidase-4 inhibitors (3%; personal communication). Therefore, sulphonylureas are probably more relevant than newer drug classes to emerging countries such as Vietnam (6, 36). Recent analyses based on systematic literature reviews have highlighted that the clinical and economic burdens of T2DM are greater in emerging markets than in established markets (37), further emphasizing the need for affordable and sustainable strategies to reduce these burdens. It is also important to point out that generic versions of gliclazide are available, which would impact the cost (and therefore cost-effectiveness) of gliclazide-based regimens. As information on the type/cost of gliclazide used in ADVANCE is not available, we used the cost of branded/originator gliclazide in our analysis.

In summary, this economic analysis showed gliclazide-based IGC to be very cost-effective compared with SGC. The findings will be informative for policymakers when making decisions on healthcare resource allocation.

## Data Availability Statement

The original contributions generated for the study are included in the article/supplementary material, further inquiries can be directed to the corresponding author.

## Author Contributions

All authors have contributed substantially to the study. OE designed the study, programmed the analysis, acquired the data from the literature, and ran the analysis. H-YN-T, NN, and NL provided experts' advice and further contributed to the acquisition of data from the literature. All authors checked assumptions ranges and interpreted the results. OE and MB drafted the manuscript that was reviewed and revised by all authors. Lastly, all authors approved the final submitted version of the manuscript.

## Conflict of Interest

The authors declare that this study received funding from Servier. Apart the required use of the ADVANCE trial publications, the funder had no influence on the design of the analysis and the choice of sources to populate it. OE had received consulting fees from Servier in the conduct of the study and the preparation of the manuscript. Editorial assistance was provided by Spirit Medical Communications Group Ltd., funded by Servier. MB was an employee of Servier. The remaining authors declare that the research was conducted in the absence of any commercial or financial relationships that could be construed as a potential conflict of interest.

## Abbreviations

ADVANCE: Action in Diabetes and Vascular Disease: Preterax and Diamicron Modified Release Controlled Evaluation
CV: cardiovascular
DRED: diabetes-related eye disease
DSA: deterministic sensitivity analysis
ESRD: end-stage renal disease
HR: hazard ratio
ICER: incremental cost-effectiveness ratio
IGC: intensive glucose control
LY: life year
MI: myocardial infarction
NA: not applicable
PSA: probabilistic sensitivity analysis
QALY: quality-adjusted life year
SGC: standard glucose control
T2DM: type 2 diabetes mellitus
USD: United States Dollars.
